# Compact Sub 6 GHz Dual Band Twelve-Element MIMO Antenna for 5G Metal-Rimmed Smartphone Applications

**DOI:** 10.3390/mi14071399

**Published:** 2023-07-09

**Authors:** Chih-Chung Lin, Shao-Hung Cheng, Shu-Chuan Chen, Cheng-Siang Wei

**Affiliations:** 1 Electrical and Electronic Engineering Department, Chung Cheng Institute of Technology, National Defense University, Taoyuan 335, Taiwan; tommylin98@gmail.com (C.-C.L.); scchen0319@gmail.com (S.-C.C.); 2 Electrical Engineering Department, National Yunlin University of Science and Technology, Douliu 640, Taiwan; rich6316@gmail.com

**Keywords:** multiple-input multiple-output (MIMO) antennas, smartphone antennas, 5G antennas, sub 6 GHz antennas

## Abstract

In this paper, a twelve-antenna system is designed for 5G smartphones with metal frames. The system is compact and operates on dual bands within the sub-6 GHz frequency range using multiple-input multiple-output (MIMO) technology. Two sets of six-antenna units are included in the system, arranged in a diagonal mirror-image configuration, and positioned at the center of the circuit board’s longer edges. The profile height of each of the six-antenna units is only 3 mm, and the overall array dimensions are 105 × 3 × 3.1 mm^3^. A single antenna unit is 15 × 3 × 3.1 mm^3^ (0.173 λ × 0.035 λ × 0.036 λ, where λ equals the free-space wavelength of 3450 MHz). The arrangement of the antennas in the six-antenna units is parallel, with a 3 mm separation between adjacent antennas. The antenna structure comprises of an inverted L-shaped feed branch and two inverted L-shaped short-circuit branches integrated into part of the metal frame. The proposed array can form multiple resonance paths, achieving dual-band operation at 3300–3600 MHz and 4800–5000 MHz. The measured isolation of this twelve-antenna system within the operating frequency band is over 10 dB, and the measured antenna efficiency is greater than 36%. Therefore, the system is suitable for use in smartphones with high screen-to-body ratios and metal frames.

## 1. Introduction

Rapid advances in communication technology have resulted in wireless communications becoming an indispensable aspect of daily life. In fifth generation (5G) mobile communications, multiple-input multiple-output (MIMO) technology is used to achieve high-speed transmission with multiple antennas. The most commonly used mobile communications devices are slim and have metallic cases, large screens, and narrow bezels. Therefore, the available space for antennas is shrinking. Correspondingly, antennas used in mobile communication devices are becoming increasingly compact with lower profiles. Designing multiple-antenna configurations within limited space to avoid excessive interference while achieving high efficiency and channel independence has become an increasingly urgent and challenging problem. Although several smartphone antenna designs using MIMO antenna arrays have been proposed in the literature [1,2,3,4,5,6,7], none have accounted for the use of metal frames. The authors of [4,5] incorporated a decoupling mechanism to improve isolation. The planar antenna configuration in [6] achieves the required self-isolation as the four antenna elements are positioned at the diagonally opposite corners of the ground plane. The antenna elements of [7] are placed along both long side edges of the mobile terminal with a low profile of 6 mm, and it exhibited good isolation without introducing the extra decoupling structure.

Smartphones with metal frames are considered to have pleasing textures and aesthetics. Moreover, they are durable and can effectively dissipate heat. Some studies [8,9,10,11,12,13,14,15,16,17,18] have designed MIMO antennas for smartphones with metal frames. In other studies, multi-antenna systems have been configured on a mobile phone device with the antennas being placed at appropriate distances from each other to optimize the isolation between the antennas [8,9,10,11,12,13]. The distances between neighboring antenna pairs are approximately 12.5–31 mm. The interaction between the two antenna elements is greatly affected by the surface current of the metal frame. To address this, the authors of [8] created a slot on a metal frame to physically separate two antenna elements and etched a groove on the ground to counteract the coupled currents. In [9], the authors proposed two types of elements, namely an inverted H-shaped slot antenna and a modified top-loaded monopole, to achieve low mutual coupling. The radiation mode of the inverted H-shaped slot antenna could not be excited by the modified top-loaded monopole, resulting in high isolation between the two antennas. The proposed antenna in [10] comprised E-shaped monopole elements that were fed by microstrip lines, thin rectangular slots in the ground plane, and multiple slots and slits in the metal frame. This design improved the mutual coupling characteristics between each antenna element without requiring a decoupling structure. In [11], the authors developed a wideband antenna pair designed for 5G mobile phones. Each element of the antenna pair features an open slot and a coupled feeding line. Grounding branches were used to minimize the mutual coupling between the two elements. In [13], the antenna elements in the authors’ design follow a slot antenna configuration, consisting of an L-shaped open slot and a 50 Ω microstrip feedline. To achieve optimal impedance matching in the higher frequency band, the authors incorporate a tuning stub loaded onto the feedline. This approach ensures excellent impedance matching and enhances the overall performance of the antenna system. Although mechanisms for achieving low mutual coupling effects have been proposed in [8,9,10,11,12,13], the designs of these studies all require a distance to be present between antenna pairs to maintain good isolation.

To design decoupling mechanisms or achieve impedance matching, integrated metal-frame MIMO antennas for smartphones have been connected with components such as capacitors or inductors [14,15,16,17,18]. In [14], the authors presented a MIMO antenna for a full-screen metal-frame smartphone that covered the low-band (824–960 MHz), mid-band (1710–2690 and 3300–3800 MHz), and high-band (5800–5825 MHz) frequencies. The low-band and mid-band antennas added capacitors and inductors as a matching circuit for impedance adjustment. In [15], the authors proposed a 2 × 2 MIMO array covering the entire long-term evolution (LTE) band (698–960 and 1710–2690 MHz) and a 4 × 4 MIMO array covering 5G bands. When the radio frequency switch (PIN diode) loaded on the LTE antenna structures was turned off, the resonance was shifted to a lower spectrum of 698–770 MHz. In addition, the LTE antenna structures included a complex matching network of capacitors and inductors that could broaden the bandwidth. In [16], the authors presented a novel dual-port single-dipole MIMO antenna pair, which used a feeding loop to selectively excite the half-wavelength dipole mode and a feeding capacitor to excite the full-wavelength dipole mode. A dual-port single-loop antenna unit between the ground plane and metal frame was proposed in [17]. To isolate the two antenna ports, a basic decoupling capacitor was employed at the center of the loop resonator. As a result, even though the two antenna ports shared a single loop structure, high port-to-port isolation was achieved. All of the MIMO antennas in these studies [14,15,16,17,18] achieved impedance matching or improved isolation. However, they included capacitors or inductors, which increased both the complexity and cost of the antenna systems.

The development of a 12 × 12 MIMO antenna for smartphones was able to improve the transmission rate, capacity, anti-interference ability, coverage, and positioning accuracy of wireless communication systems, thereby improving the performance and user experience of wireless communication [19,20,21,22,23,24]. A twelve-element inverted-F antenna array for smartphones was constructed in [19]. The array used the hybrid decoupling method. Multiple decoupling capacitors were used to couple two back-to-back inverted-F antenna pairs and T-shaped mode transformers for the other couplings. A hybrid twelve-element MIMO antenna array for 5G smartphones was presented in [21]. The proposed antenna comprised four identical three-element antenna arrays. Two L-shaped monopole slots on the ground were placed symmetrically on both sides of each M-shaped loop antenna. Because these two antenna types had orthogonal polarization, coupling was reduced. In [21], the authors proposed a dual-band twelve-element MIMO antenna system with a T-shaped slot for each antenna. They applied spatial diversity and orthogonal antenna placement (i.e., feeding strips of two antennas are not pointing at each other) to improve isolation. However, none of the proposed twelve-element MIMO antenna systems of these studies [19,20,21,22,23,24] have been verified for use in smartphones with metal frames.

An antenna configuration must be compact, should be effective in a metal frame, and should achieve high throughput by using multiple antennas. We therefore propose a compact configuration for a 5G dual-band 12 × 12 MIMO antenna system that is integrated with the metal frame on a smartphone. The first feature of the proposed compact antenna design is a distance between two elements of only 3 mm. Both sides of each element have short-circuit branches, which reduces the impact of the ground plane current on adjacent antennas. Therefore, the proposed antenna design can achieve good isolation without decoupling elements and has narrow spacing between elements of only 3 mm. The second feature is the structural design of an inverted L-shaped feeding branch and two inverted L-shaped short-circuiting branches in the clearance area. Because the length of the inverted L-shaped feeding branch and the configuration of the two inverted L-shaped short-circuiting branches were adjusted, the operating frequency bands were impedance matched at 3300–3600 MHz and 4800–5000 MHz. The third feature is that the profile height of the antenna is only 3 mm, enabling it to fit in a narrow frame. The resonance path covers part of the metal frame, and the opening position of the metal frame is used to excite the low and high-frequency resonance paths. The proposed antenna system has an envelope correlation coefficient (ECC) of less than 0.38 and an isolation greater than 10 dB without any isolation components, and its efficiency is greater than 36%.

This paper is structured in the following manner. We first present the design details of the MIMO antenna system in the following section. The design mechanism and parametric analyses are discussed in Section 3. Afterward, Section 4 presents a discussion of the radiation performance metrics of the antenna, such as its reflection coefficients, transmission coefficients, ECC, efficiency, and radiation pattern. The MIMO channel capacity are discussed in Section 5, and Section 6 presents an analysis of the characteristics of the MIMO antenna system. In Section 7, we present the conclusion.

## 2. Proposed MIMO Antenna System Design

**Figure 1:**
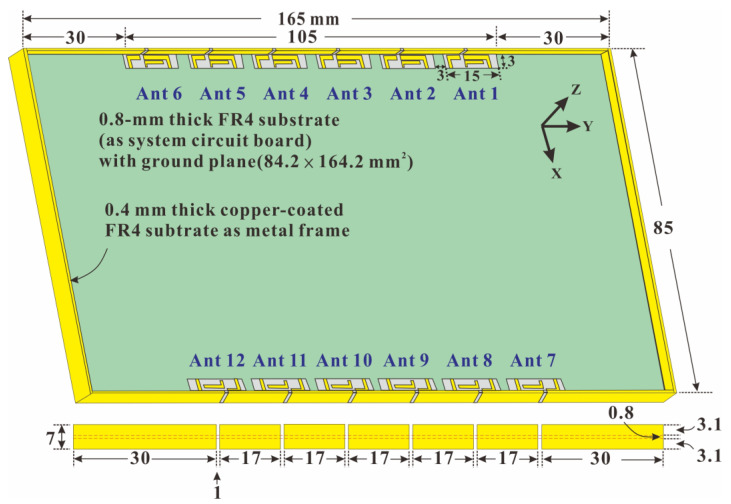
Overall configuration of the sub-6-GHz 5G MIMO twelve-antenna system.

Figure 1 presents the overall configuration of the proposed sub-6-GHz MIMO twelve-antenna system. The system was designed for a 6.8-inch smartphone with a thickness of 7 mm and overall dimensions of 165 × 85 × 7 mm^3^. The system circuit board is positioned at the center of the metal frame. The board comprises an FR4 glass fiber substrate with the dimensions of 84.2 × 164.2 × 0.8 mm^3^. The metal frame consists of a 0.4 mm thick copper-coated FR4 glass fiber substrate with a 7 mm width. The FR4 substrate has a relative dielectric constant of 4.4 and a loss tangent of 0.02. The dual-band MIMO twelve-antenna units (Ant 1, Ant 2, Ant 3, Ant 4, Ant 5, Ant 6, Ant 7, Ant 8, Ant 9, Ant 10, Ant 11, and Ant 12) are arranged into two sets of six-antenna units, which are diagonally and mirror-symmetrically arranged on both sides of the system circuit board and centered on the long side edge at a distance of 30 mm from the short edges of the phone. The profile height of each antenna element is only 3 mm, and the dimensions of the antenna elements are 105 × 3 × 3.1 mm^3^. All six-antenna units have the same structure and size and are arranged in parallel at a distance of 3 mm from their neighbors.

This design not only integrates part of the metal frame as part of the antenna resonance path but also uses a specifically designed short-circuit branch on both sides of each antenna structure, which is coupled with the opening position of the metal frame, to excite the low and high-frequency resonance paths on both sides. By arranging the six-antenna elements of each unit in a parallel and codirectional manner with 3 mm spacing, we isolate the low and high-frequency resonance paths between adjacent antennas. Additionally, regardless of whether the low and high-frequency modes are excited, the short-circuit branches can attract a ground plane current, which effectively reduces the flow of the current to adjacent antennas. This design achieves good isolation performance with a spacing of only 3 mm between the antennas without requiring isolation components.

**Figure 2:**
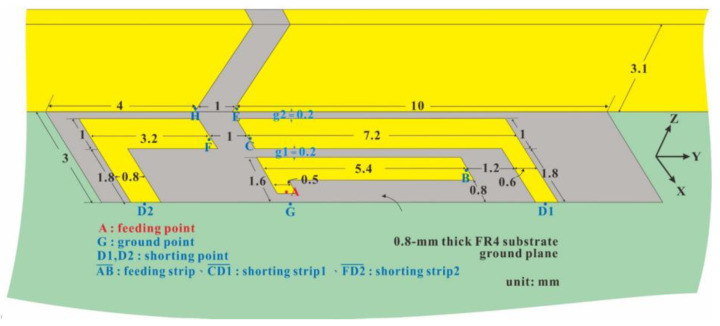
Detailed structure of an antenna element in the sub-6-GHz 5G MIMO system.

Figure 2 presents the detailed structure of a sub-6-GHz antenna element of the proposed system. All of the twelve-antenna units have the same structure and size. The antenna dimensions are 15 × 3 × 3.1 mm^3^, and the antennas are configured on the system circuit. Each antenna has a clearance zone without a ground plane with a size of 15 × 3 mm^2^. The clearance zone features a reversed L-shaped feed branch and two reversed L-shaped short-circuit branches. It is integrated with the upper metal frame to form multiple resonant paths for dual-frequency band sub-6-GHz operation (i.e., 3300–3600 MHz and 4800–5000 MHz). The metal frame has a 1 mm opening above the clearance zone at 4 mm and 10 mm from the left and right ground planes, respectively. This antenna structure has both low and high-frequency resonance modes covering the dual-frequency band operation of 3300–3600 MHz and 4800–5000 MHz. The low-frequency resonance is enhanced by the dual-coupled loop path formed by the feeding strip ($\bar{AB}$), which is coupled to shorting strip 1 ($\bar{CD1}$) and then coupled to the upper edge of the metal frame. The high-frequency resonance is enhanced by the loop path formed by left shorting strip 2 ($\bar{FD2}$), which is coupled to the upper edge of the metal frame. Point A in Figure 2 is the feeding point, which is connected to the inner conductor of the 50-Ω coaxial transmission line (50-Ω mini coaxial line), and the outer conductor of the 50-Ω coaxial transmission line is connected to ground shorting point G on the metal ground plane of the screen.

## 3. Antenna Element Design Mechanism and Parameter Analysis

**Figure 3:**
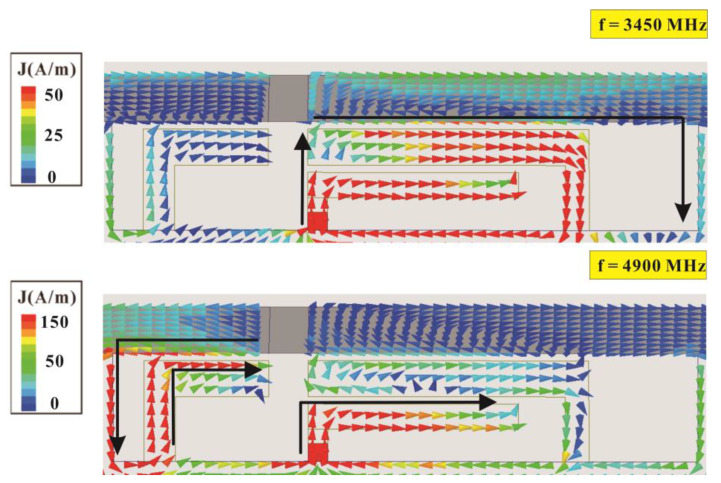
Surface current distribution of an antenna element at 3450 MHz and 4900 MHz.

To verify the operating principle of the antenna element and analyze the contribution paths of each mode within the operating band, we analyze the surface current distribution of the sub-6-GHz antenna element at the resonance frequency points in the low-frequency band (3450 MHz) and high-frequency band (4900 MHz). Figure 3 presents the surface current distributions of a single sub-6-GHz antenna element at 3450 MHz and 4900 MHz. At 3450 MHz, the current on the right side of the antenna is greater than that at 4900 MHz and is mainly distributed in the double-coupling loop comprising the feeding strip ($\bar{AB}$) coupled to shorting strip 1 ($\bar{CD1}$) and coupled to the upper edge of the metal frame on the right side. Therefore, the low-frequency resonance mode is mainly achieved through this right-side loop path. At 4900 MHz, the current is mainly distributed in the loop path formed by the feeding strip ($\bar{AB}$) and left shorting strip 2 ($\bar{FD2}$), which is coupled to the upper edge of the metal frame on the left side. Therefore, the high-frequency resonance mode is mainly achieved by this left-side loop path.

**Figure 4:**
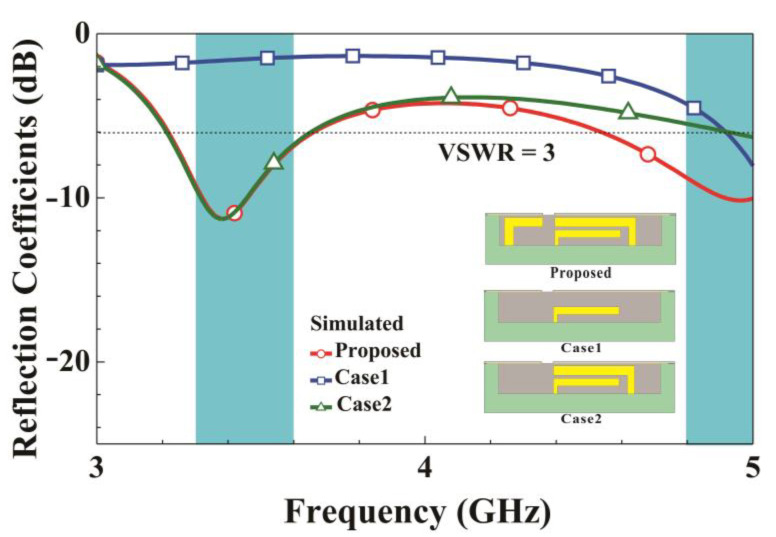
Simulated reflection coefficients for the proposed system, Case 1, and Case 2.

To further understand the contribution of each mode, we analyze the proposed structure and two other designs denoted Case 1 and Case 2. Case 1 is the proposed antenna structure without shorting strip 1 ($\bar{CD1}$) and shorting strip 2 ($\bar{FD2}$), and Case 2 is the proposed antenna structure with shorting strip 1 ($\bar{CD1}$). Figure 4 presents a comparison of the simulated reflection coefficients for Case 1, Case 2, and the proposed structure. The Case 1 antenna structure does not produce any resonant modes in the low-frequency band but has a shallow resonant mode near 5000 MHz in the high-frequency band. When shorting strip 1 ($\bar{CD1}$) is added to the Case 1 antenna structure to form Case 2, a favorable resonant mode is generated in the low-frequency band. However, the high-frequency resonant mode matching is impaired. By adding a shorting strip 2 ($\bar{FD2}$) to form the proposed structure, the low-frequency mode can be preserved, and the high-frequency mode can be further optimized to cover the desired 4800–5000 MHz frequency band. The low-frequency resonant mode is mainly enhanced by the double-coupling loop path formed by the feeding strip ($\bar{AB}$) coupled to shorting strip 1 ($\bar{CD1}$) and then to the upper metal edge of the structure. The high-frequency mode is mainly enhanced by the left loop path formed by the feeding strip ($\bar{AB}$) and the left shorting strip 2 ($\bar{FD2}$) coupled to the metal frame at its upper edge.

**Figure 5:**
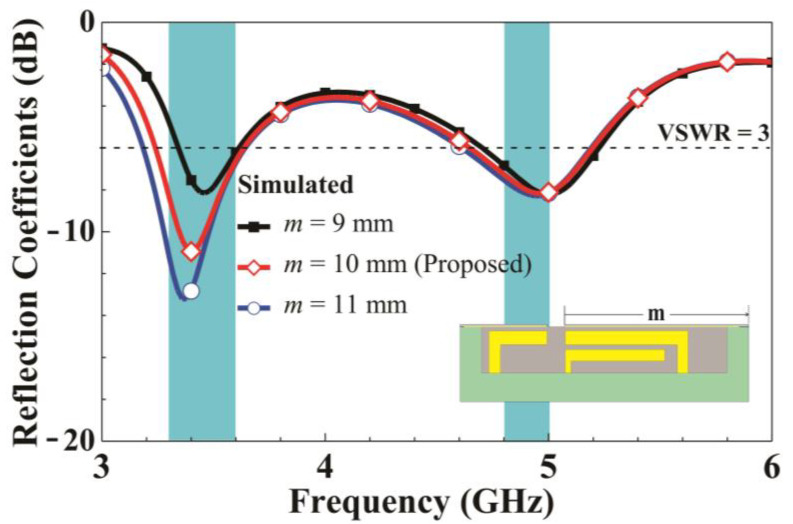
Simulated reflection coefficients for various *m* values.

Three parameter analyses are conducted for the structure of Ant 1. First, a parameter analysis for length m of the right side of the metal frame with a 1 mm opening on the metal frame is conducted. The opening position is fixed, and grounded metal is added or removed at an appropriate clearance interval to increase or decrease the effective length of the right metal frame. Figure 5 presents a comparison of the simulated reflection coefficients as *m* varies. Increasing the right-side length of the metal frame from 9 to 11 mm generates a substantial downshift in the low-frequency resonant mode but does not affect the high-frequency resonant mode. This verifies that the low-frequency resonant mode is contributed to by the path on the right side of the metal frame opening. A parameter analysis for length *n* of the left side of the metal frame with a 1 mm opening is then conducted. The opening position is fixed, and grounded metal is added or removed as appropriate in the left clearance interval.

**Figure 6:**
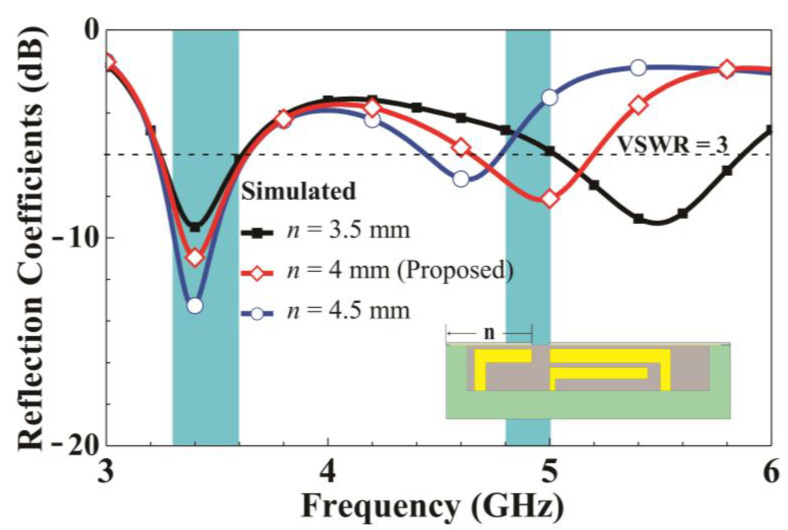
Simulated reflection coefficients for various *n* values.

**Figure 7:**
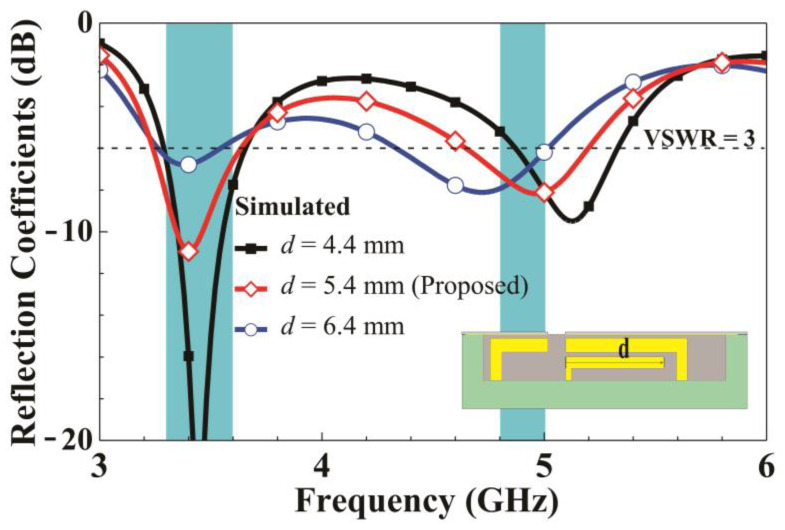
Simulated reflection coefficients for various *d* values.

Figure 6 presents the simulated reflection coefficients as n changes. When the left-side length of the metal frame increases from 3.5 to 4.5 mm, the low-frequency matching changes only slightly. However, the high-frequency resonant mode exhibits a substantial downshift as *n* increases. This is because when *n* increases, the length of the loop path coupled to the metal frame (left shorting strip 2) increases. This result demonstrates that the left loop path contributes to the high-frequency mode. Finally, parameter analysis for the end length d of the inverted L-shaped feeding branch is conducted. Figure 7 presents the simulated reflection coefficient for various *d* values. Increasing *d* from 4.4 to 6.4 mm leads to a substantial downshift in both the low and high-frequency resonant modes. Therefore, the feeding strip path contributes to both resonant modes.

## 4. Experiment and Measurement Results

**Figure 8:**
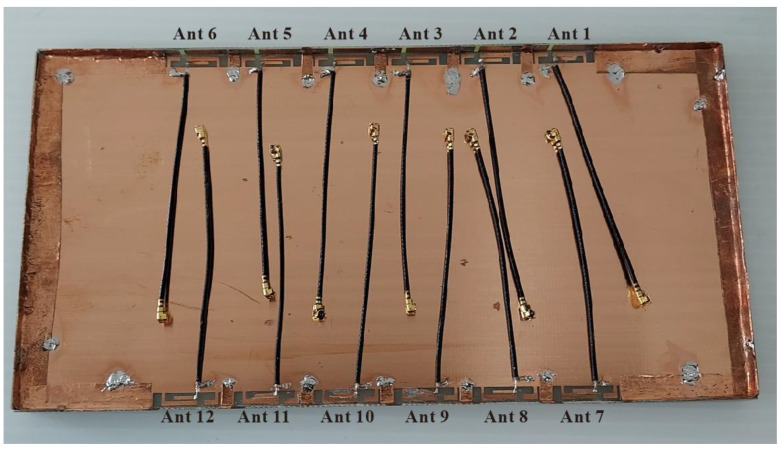
Fabricated MIMO twelve-antenna system.

**Figure 9:**
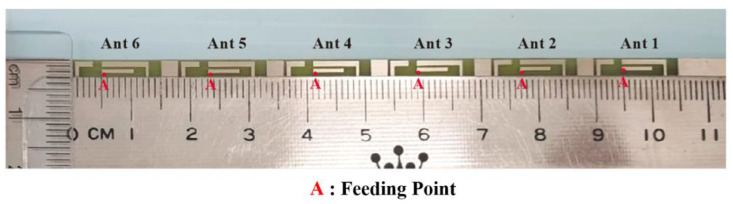
Fabricated MIMO six-antenna unit.

Figure 8 presents the overall configuration of the fabricated MIMO twelve-antenna system. Figure 9 displays the fabricated MIMO six-antenna unit and the antenna elements in detail. The antennas were fabricated in accordance with the dimensions presented in Figure 1 and Figure 2 and were measured. Ansys HFSS (Version 18) software was used to conduct simulations. In the simulations, the desired threshold for the reflection coefficient was set to be less than -6 dB (equivalent to a voltage standing wave ratio of 3:1) [25,26,27]. This means that if the reflection coefficient of the antenna in both simulation and measurement was below -6 dB, it indicates that the desired antenna efficiency metrics could be easily achieved in actual tests. The transmission coefficient between the antennas was set as less than −10 dB on the basis of the findings of previous studies [25,26,27].

**Figure 10:**
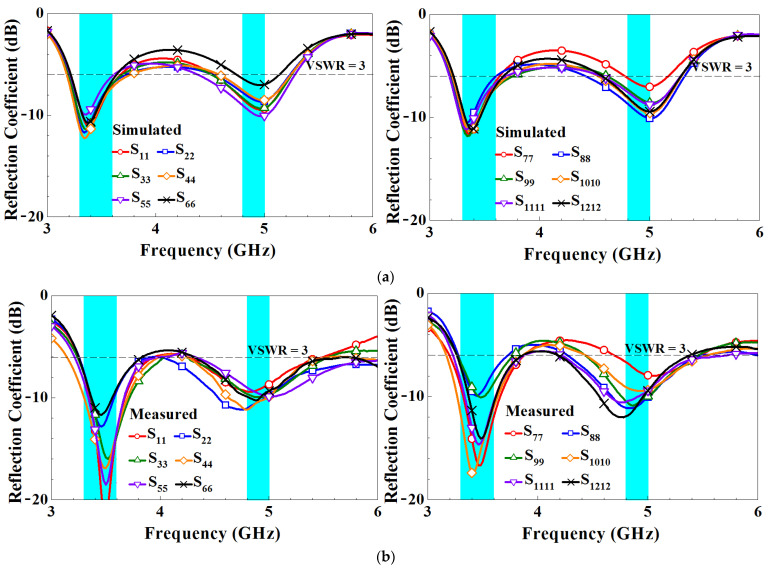
(**a**) Simulated and (**b**) measured reflection coefficients of the MIMO twelve-antenna array.

Figure 10 presents the simulated and measured reflection coefficients of the MIMO twelve-antenna system. Both the simulated and measured reflection coefficients indicate that the system can achieve dual-band 5G sub-6-GHz operation at 3300–3600 MHz and 4800–5000 MHz. Both the low and high-frequency bands have one resonance mode, and the simulated and measured data are consistent. Moreover, both the simulated and measured reflection coefficients are smaller than −6 dB (i.e., voltage standing wave ratio = 3:1).

**Figure 11:**
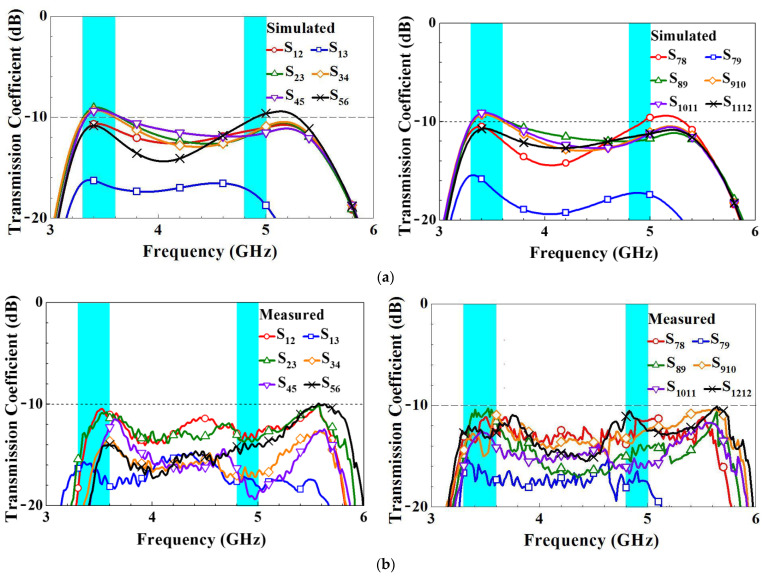
(**a**) Simulated and (**b**) measured transmission coefficients of the MIMO twelve-antenna array.

Figure 11 presents the simulated and measured transmission coefficients of the MIMO twelve-antenna system. For clarity, we only present the transmission coefficients of two adjacent antennas because adjacent antennas have the greatest mutual influence. The transmission coefficients for antenna pairs with greater separation are smaller [25]. The figure reveals that not all simulated transmission coefficients are smaller than −10 dB in the frequency band (only −9.5 dB or higher was achieved). However, the actual measurements demonstrate that transmission coefficients of -10 dB or better were achieved. In antenna design, measured data is often considered a more reliable basis than simulated data. This is because measured data reflect true performance under actual manufacturing and operating conditions. While simulations can provide valuable reference and initial assessments, they are still based on assumptions and idealized models. In Figure 10 and Figure 11, measured results slightly differ from and improve compared to the simulated ones. These discrepancies and causes could be due to the fact that the additional mini coaxial cables and IPEX-to-SMA adaptors used in measurement causing loss are not considered in simulation. Moreover, the parameter variation of the FR4 substrate and the imperfection in fabrication could also be part of the reasons for the discrepancies.

**Figure 12:**
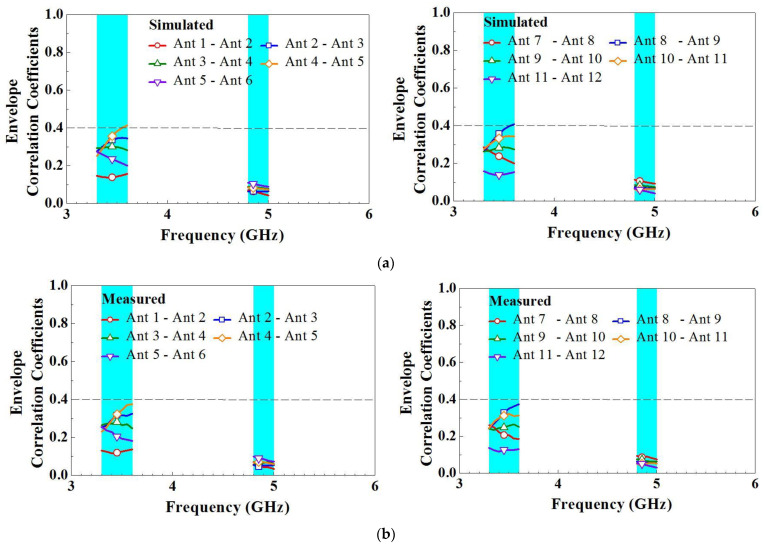
ECCs of the (**a**) simulated and (**b**) measured radiation patterns of the twelve-antenna system.

**Figure 13:**
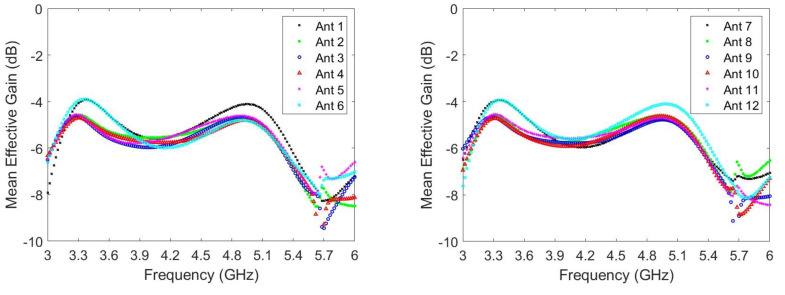
MEGs of the twelve-antenna system.

**Figure 14:**
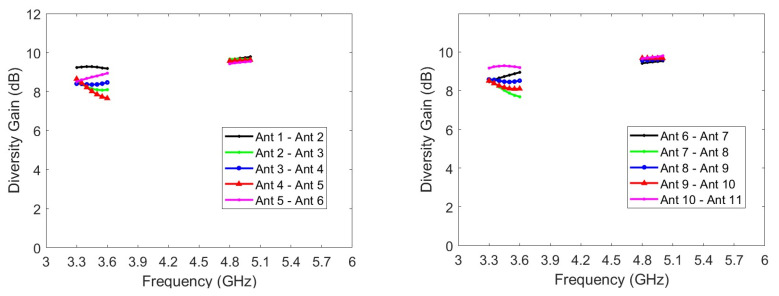
DGs of the twelve-antenna system.

For MIMO operations, certain performance parameters such as the ECC, mean effective gain (MEG), diversity gain (DG), and total active reflection coefficient (TARC) may also be of interest and can be examined. The ECC is a critical metric for evaluating MIMO antenna systems. Generally, an ECC of less than 0.5 is satisfactory for practical applications. Figure 12 presents the ECCs of the simulated and measured radiation pattern for the MIMO twelve-antenna system. For clarity, only the ECCs of adjacent antennas are presented because the influence is greatest for adjacent antennas. The simulated and measured ECCs within the frequency bands are all less than 0.41 and 0.38, respectively. Moreover, the trends of the simulated and measured ECC variations are in favorable agreement. Although the twelve-antenna units have the same structures, the ECC values between the antenna pairs (i.e., Ant 1–Ant 2, Ant 2–Ant 3, and Ant 3–Ant 4) differ because of the different ground plane positions adjacent to the antennas. However, all ECCs are sufficiently low for practical applications. Therefore, the antennas can be considered to have favorable channel independence. MEG is a crucial parameter in diversity power analysis, representing the ratio of the average power absorbed by the antenna to the average incident power of the antenna system. This serves as an important metric in evaluating the efficiency and performance of the antenna system. In the lower frequency band, the MEGs range from −3.90 to −5.57 dBi, while in the higher frequency band, they range from −4.10 to −4.92 dBi, as shown in Figure 13. It is worth noting that the maximum difference in excited MEGs between Ant-3 and Ant-6 is only 1.10 dB, occurring at 3.48 GHz. This difference is considered small enough for practical applications. In addition, DG is a significant parameter in MIMO systems as it measures the reduction in transmitted power loss achieved through the implementation of diversity mechanisms. Figure 14 displays the DG curves of the proposed MIMO antenna. The antenna system exhibits a diversity gain greater than 7.66 dB at low frequencies and 9.43 dB at high frequencies.

**Figure 15:**
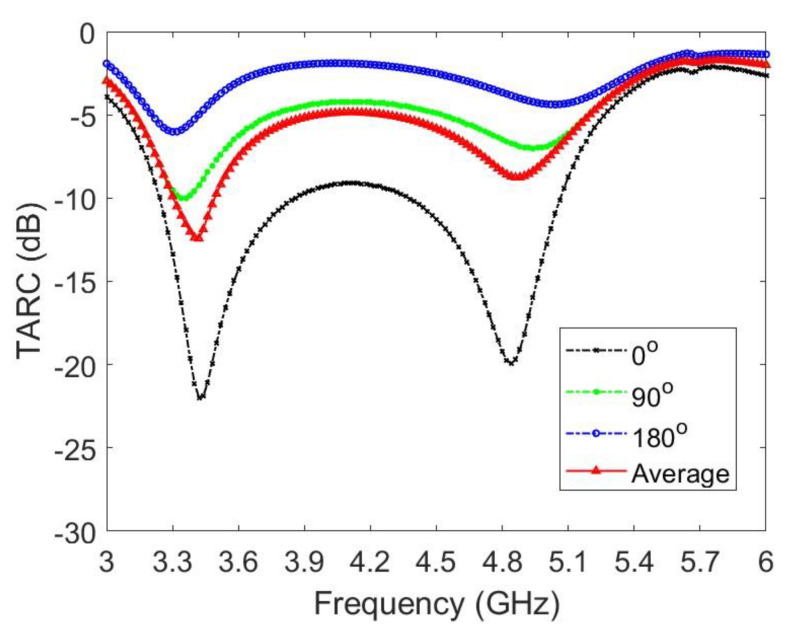
TARCs for the proposed twelve-antenna system.

Next, TARC is the square root of the incident power obtained from all excitations minus the radiant power, divided by the incident power. To maintain clarity and avoid a cluttered appearance, Figure 15 presents TARC data for only three specific values of θ: 0°, 90°, and 180°. However, the average TARC, calculated by averaging over the range of θ from 0° to 180° with a sampling spacing of 9°, consistently remains below −7.5 dB within the desired frequency range. This value is smaller than the -6 dB threshold for the impedance band.

**Figure 16:**
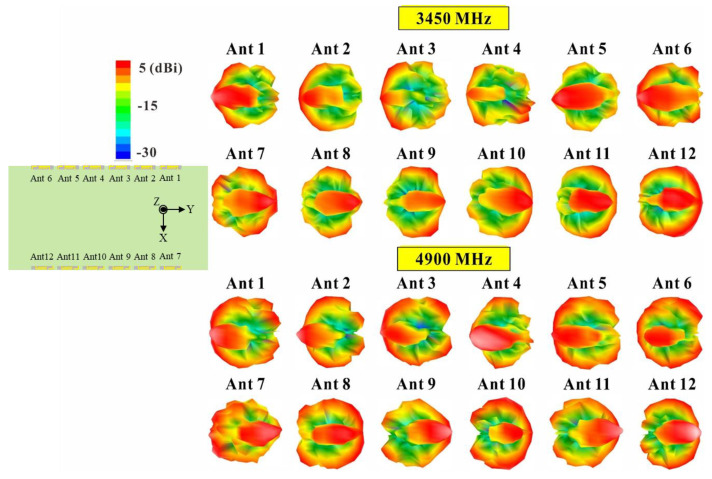
Measured 3D radiation patterns of the twelve-antenna units at 3450 MHz and 4900 MHz.

Figure 16 presents the three-dimensional (3D) radiation patterns for each antenna at 3600 MHz and 4800 MHz. The far-field radiation patterns of the twelve-antenna units operating at 3600 MHz and 4800 MHz were found to be similar but not identical. The 3D radiation pattern provides a full range of radiation performance information, including the radiation characteristics of the antenna in the horizontal (H-plane) and vertical (E-plane) directions. The computed ECC values from the measured radiation patterns were all less than 0.38. This result is also consistent with the ECC values shown in Figure 7. It can also be observed that the two sets of six-antenna units each are configured in a slanting mirror image arrangement, which results in a slanting mirror image characteristic in their radiation patterns.

**Figure 17:**
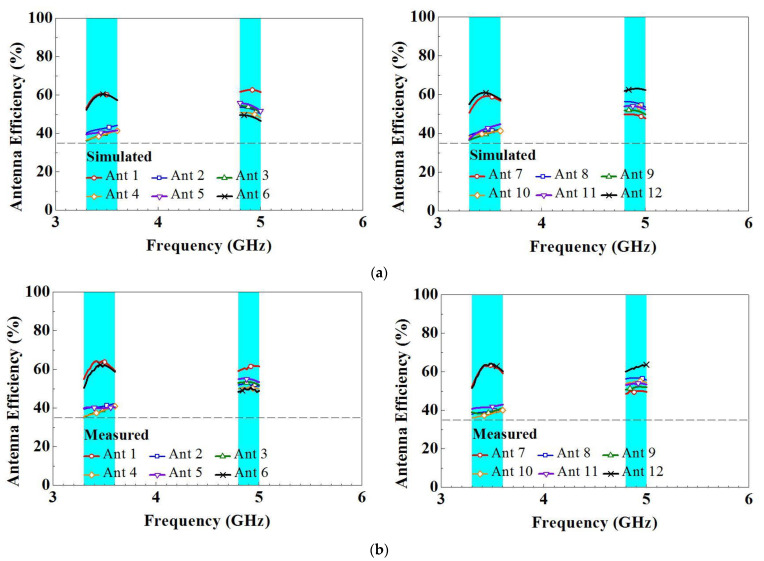
(**a**) Simulated and (**b**) measured antenna efficiency for the twelve-antenna system.

Figure 17 presents the simulated and measured antenna efficiency of the system. The simulated and measured efficiencies for each frequency band are both greater than 36%, indicating good performance. The measured efficiency values of the high- and low-frequency bands are 36–62% and 46–63%, respectively. The simulation and measurement data have a slight discrepancy. This may be attributable to the transmission line and its length, which were not included in the simulation. Moreover, the soldering, FR4 substrate, and copper sheet used to fabricate the antennas may not have been accurately reproduced in the simulation. In the literature focusing on the integration of metal frame MIMO mobile phone antennas, the radiation efficiency was reported to range from 31% to 65% at 3300 MHz–3800 MHz (with a 50.4 mm spacing between the antennas) [15], and the radiation efficiency was reported to range from 36% to 58% at 4800 MHz–5000 MHz (with a 21 mm spacing between the antennas) [14]. It is important to note that metal frames can have an impact on radiation efficiency due to their ability to reflect, absorb, or scatter RF energy, resulting in radiation loss or non-uniform radiation patterns. Considering these factors, we propose that a radiation efficiency of over 36% is sufficient for the dual-band MIMO mobile phone antenna integrated with a metal frame, especially when the antennas are positioned with a mere 3 mm spacing between them. This level of efficiency is expected to meet the communication requirements and provide a good connection range and stability.

## 5. MIMO Channel Capacity

The ergodic channel capacity refers to the average capacity observed in a multipath Rayleigh fading environment. In cases where a transmitter lacks channel state information (CSI) and its power is evenly distributed among each transmit antenna unit, the ergodic channel capacity can be calculated using the following formula [25]:

(1) $$C=E\{{log}_{2}[det(I+\frac{S}{n_{t}}HH^{†})]\}$$

where *E* is the expectation concerning different channel realizations, *I* is the identity matrix, det is the determinant, *s* is the signal-to-noise ratio (SNR), *n_t_* is the number of transmit antennas, *H* is the wireless channel matrix, and ()^†^ is the Hermitian transpose of the matrix.

**Figure 18:**
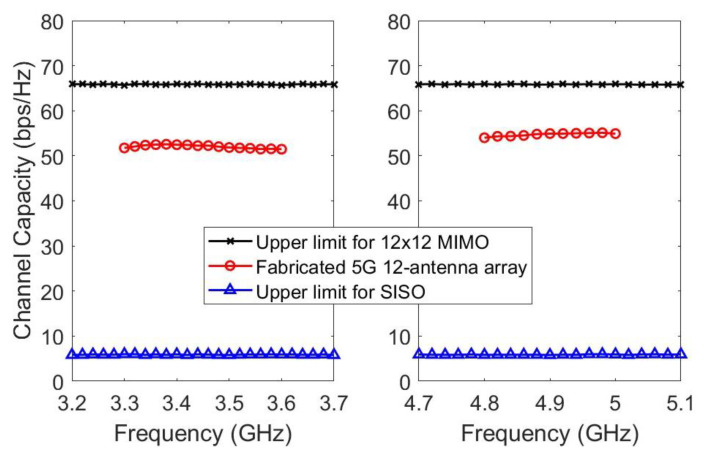
Calculated ergodic channel capacities of 5G antenna array prototype in 12 × 12 MIMO system.

Figure 18 illustrates the results of the calculated ergodic capacity at a 20 dB signal-to-noise ratio (SNR). The performance of the proposed 5G twelve-antenna array in free space is compared to an ideal 12 × 12 MIMO system and a single-input single-output (SISO) system. In the comparison, the ideal transmitting and receiving antennas possess 100% efficiency, null ECC, and independent and identically distributed (i.i.d.) channels with Rayleigh fading. To ensure convergence to specific values, the capacity results are obtained by averaging 1,000 channel realizations at each frequency point. In the free space scenario, the capacity within the 3300–3600 MHz range ranged from 51.48 to 52.58 bit/s/Hz, while in the 4800–5000 MHz range, it ranged from 53.92 to 55.11 bit/s/Hz. Notably, the highest capacity value in the 3300–3600 MHz band was 10 bit/s/Hz lower than the capacity of the 12 × 12 MIMO i.i.d. channels. The proposed twelve-antenna array demonstrates a significantly larger capacity compared to a SISO system, making it a promising solution for future 5G laptop applications.

## 6. Analysis of the MIMO Antenna System and Advantages

**Figure 19:**
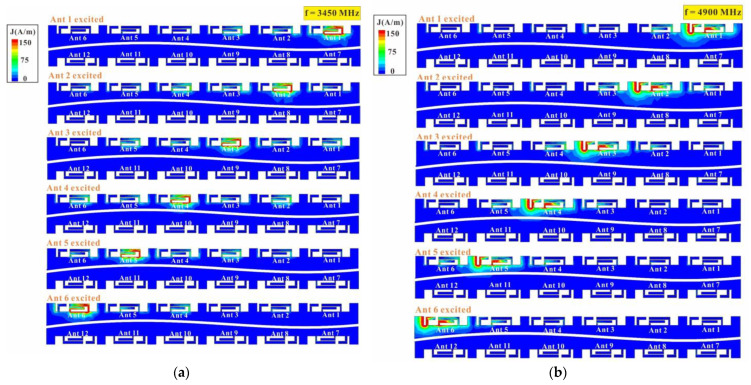
Current distribution of the twelve-antenna system and ground plane at (**a**) 3450 MHz and (**b**) 4900 MHz.

To further investigate the isolation performance of the MIMO twelve-antenna unit, the surface current distribution between the two sets of six-antenna units and their ground planes is analyzed. The two sets of six-antenna units are configured in a diagonal mirror-image configuration. For clarity, only the surface current distribution of the first set of six-antenna when excited is presented. Figure 19 presents the surface current distribution of the two groups of six-antenna units and their ground planes at 3450 MHz and 4900 MHz. The results reveal that regardless of which antenna is excited, no noticeable ground current flows to other antennas are observed. The surface current distribution is smaller for antennas further away from the excited antenna. Therefore, this twelve-antenna unit has favorable isolation performance.

To emphasize the benefits of our suggested MIMO antennas, we have incorporated a comparative analysis of MIMO antennas from the existing literature. Table 1 presents this comparison, encompassing various aspects such as dimensions, spacing between two-antenna units, isolation, and radiation performance for 5G sub-6GHz band. Upon reviewing the table, it becomes apparent that our proposed MIMO antennas exhibit a more compact design in comparison to those in the existing literature, particularly with regard to multi-band frequencies. The distance between elements of our proposed antenna system is significantly lower (only 3 mm) compared to most existing state-of-the-art solutions. The presence of short-circuit branches can generate ground plane currents, effectively reducing the current flow to adjacent borehole antennas. This design achieves greater than 10 dB isolation performance (no additional antenna isolation components required) and sufficient radiation efficiency and ECC performance.

## 7. Conclusions

The proposed compactly configured a dual-band MIMO twelve-antenna system with a metal frame comprising two sets of six-antenna units arranged in a diagonal mirror-image configuration at the edges of the two longer sides of a phone. The profile height of each of six-antenna module is only 3 mm. The design has no isolation components but is able to achieve favorable isolation and ECC performance with only 3 mm spacing between adjacent antennas. In the designing of the sub-6GHz 5G antenna element, a metal-frame design was integrated with an inverted L-shaped feed branch and two inverted L-shaped short branch structures to form multiple resonant paths, which enabled dual-band operation in the 3300–3600 and 4800–5000 MHz 5G frequency bands. The antenna structure is simple and can be closely integrated with the metal environment. The isolation performance values for this MIMO twelve-antenna system are all above 10 dB, and the measured far-field radiation pattern ECC values are all less than 0.38. The low-frequency efficiency is 36–62%, and the high-frequency efficiency is 46–63%. This design is suitable for large-screen smartphones with a high screen-to-body ratio and metal frame.

## Figures and Tables

**Table 1:** Performance comparison of MIMO antenna systems.

Ref.	Total Size (mm^3^)	Element Size (mm^2^)	Metal-Frame	Operating Bands (MHz)	Spacing between Two-Antenna Units (mm)	Isolation (dB)	Eff.	ECC	MIMO Order
[8]	155 × 76 × 7.5	6.78 × 6.7	Yes	4400–5000	≥31	≥22	≥50%	≤0.049	8
[9]	145 × 75 × 6	29 × 8.75 10 × 5	Yes	3300–3600	≥18	≥ 20	≥49.4%	≤0.013	8
[10]	150 × 75 × 6	11.4 × 8.4	Yes	3300–6000	≥22	≥12.8	≥50%	≤0.06	8
[11]	147 × 76 × 7	14.5 × 1.5	Yes	3300–6000	≥12.5	≥11.4	≥46%	≤0.26	8
[14]	158 × 72 × 7	59.2 × 2 40 × 2 18.5 × 2	Yes	824–960 1710–3800 4800–5825	≥21	≥ 15	≥31%	≤0.3	2 2 4
[15]	158 × 73 × 7	56.7 × 25.3 15 × 25.3 14.55 × 2	Yes	698–960 1710–2690 3300–4500	≥50.4	N/A	≥17%	≤0.48	2 2 4
[16]	140.6 × 70.6 × 6	28 × 2	Yes	3300–3800	Shared dipole	≥16	≥68%	≤0.1	4
[17]	140.6 × 70.6 × 6	67 × 2	Yes	3400–3600	Shared loop	≥16	≥55%	≤0.1	4
[18]	150 × 75 × 7.5	29 × 3	Yes	3400–3600	≥30	≥24	N/A	N/A	4
[19]	160 × 80 × 7	14.5 × 1.5	No	3300–3800	≥11.5	≥10	≥58%	≤0.2	12
[20]	150 × 75 × 7	20 × 6.5 8.5 × 6.2	No	3400–3600	≥8	≥10	≥61%	≤0.15	12
[21]	150 × 80 × 0.8	18.2 × 7.5	No	3400–3800	≥9.1	≥14.8	≥74%	≤0.05	12
Proposed	165 × 85 × 7	15 × 3	Yes	3300–3600 4800–5000	3	≥10	≥36%	≤0.38	12

## Data Availability

Not applicable.
